# Addressing colon cancer patients’ needs during follow-up consultations at the outpatient clinic: a multicenter qualitative observational study

**DOI:** 10.1007/s00520-022-07222-z

**Published:** 2022-06-21

**Authors:** Julien A. M. Vos, Laura A. M. Duineveld, Vera E. van Miltenburg, Inge Henselmans, Henk C. P. M. van Weert, Kristel M. van Asselt

**Affiliations:** 1 Department of General Practice, Amsterdam UMC, location University of Amsterdam, Meibergdreef 9, Amsterdam, The Netherlands; 2 Amsterdam Public Health Research Institute, Amsterdam, The Netherlands; 3 Cancer Centre Amsterdam, Amsterdam, The Netherlands; 4 Department of Medical Psychology, Amsterdam UMC location University of Amsterdam, Meibergdreef 9, Amsterdam, The Netherlands

**Keywords:** Colonic neoplasms, Aftercare, Survivorship, Cancer follow-up, Qualitative research, Observation

## Abstract

**Purpose:**

To describe colon cancer patients’ needs and how healthcare providers respond to these needs during routine follow-up consultations in hospital.

**Methods:**

A multicenter qualitative observational study, consisting of follow-up consultations by surgeons and specialized oncology nurses. Consultations were analyzed according to Verona Coding Definitions of Emotional Sequences. Patients’ questions, cues, and concerns were derived from the data and categorized into supportive care domains. Responses of healthcare providers were defined as providing or reducing space for disclosure. Patient satisfaction with care was measured with a short questionnaire.

**Results:**

Consultations with 30 patients were observed. Questions typically centered around the health system and information domain (i.e., follow-up schedule and test results; 92%). Cues and concerns were mostly associated with the physical and daily living domain (i.e., experiencing symptoms and difficulties resuming daily routine; 43%), followed by health system and information (i.e., miscommunication or lack of clarity about follow-up; 28%), and psychological domain (i.e., fear of recurrence and complications; 28%). Problems in the sexuality domain hardly ever arose (0%). Healthcare providers provided space to talk about half of the cues and concerns (54%). Responses to cancer-related versus unrelated problems were similar. Overall, the patients were satisfied with the information and communication received.

**Conclusions:**

Colon cancer patients express various needs during consultations. Healthcare providers respond to different types of needs in a similar fashion. We encourage clinicians to discuss all supportive care domains, including sexuality, and provide space for further disclosure. General practitioners are trained to provide holistic care and could play a greater role.

**Supplementary Information:**

The online version contains supplementary material available at 10.1007/s00520-022-07222-z.

## Background

Worldwide, the population of colon cancer patients is growing [1]. After initial treatment, colon cancer patients enter a survivorship care program for 5 years, with emphasis on the detection of recurrences (follow-up), management of the repercussions of cancer, and rehabilitation (aftercare) [2]. Many patients experience persistent symptoms long after treatment completion [3, 4] and contact both their oncology specialist and general practitioner (GP) with their questions and problems relating to colon cancer or its treatment [5–7]. Healthcare providers have different strategies for dealing with the repercussions of cancer and for attending to their patients’ needs [8].

In the Netherlands, survivorship care for colon cancer patients is provided in hospital by a specialist. Over the past few years, there have been calls for increased GP involvement [9]. The holistic approach of GPs and their familiarity with a patient’s history and psychosocial context could facilitate the provision of personalized survivorship care [10, 11], which could improve post-treatment quality of life [12, 13], patient satisfaction with care, and the perceived quality of care [14, 15]. The provision of cancer survivorship care by a GP has similar clinical and patient-reported outcomes as that provided by a specialist [16, 17]. In order to assess the role and potential added value of increased GP involvement, it is important to understand the type of problems patients experience during follow-up, and how these problems are addressed by their healthcare providers. Routine follow-up consultations at the outpatient clinic provide an opportunity to identify and address patients’ needs on a structural basis.

The primary objective of this study was to describe colon cancer patients’ needs and how healthcare providers respond to these needs during routine follow-up consultations in hospital. A secondary objective was to assess patients’ satisfaction with information and communication in order to gain a comprehensive insight into the extent to which patients’ needs are addressed. These findings may facilitate discussion of the role and potential added value of increased GP involvement in the follow-up care of colon cancer patients.

## Methods

### Design and study population

This multicenter qualitative observational study was carried out at the outpatient surgery clinic of five Dutch hospitals (one academic medical center and four community-based hospitals). The study focused on colon cancer patients during the first 5 years after primary treatment. The patients who had undergone surgery for colon carcinoma stages I–III and who had received routine follow-up care according to the Dutch national guideline were deemed eligible and included consecutively [18]. The patients were excluded if they did not speak Dutch or if they were currently receiving cancer treatment. In the Netherlands, routine follow-up consultations for colon cancer patients are performed by surgeons, and in some cases by specialized oncology nurses working under supervision of a surgeon. A specialized oncology nurse is a registered nurse who is specialized in providing care for cancer patients and their families. These nurses coordinate care to help ensure that cancer patients’ needs are met.

### Study procedure

Prior to the routine follow-up consultations, the patients were informed about the study procedure, and written informed consent was obtained by the observers (Ms. FA and Ms. TvdK). Observers did not participate in the dialogue between the patient and healthcare provider. Follow-up consultations were audio-recorded and transcribed verbatim. Transcripts were verified against audio-data and anonymized (Ms. LD and Mr. JV). Consultations lasted between 02:14 and 24:54 min (median of 6 min, IQR 5 min). The consultation which lasted only 02:14 min was with a female patient, age 52, who had stage III colon carcinoma and who had been followed up for 18 months On average, consultations with a surgeon were shorter (median 6 min, IQR 3 min) than consultations with a specialized oncology nurse (median 15 min, IQR 3 min). Qualitative findings were considered sufficient when the transcripts provided no new types of questions/cues/concerns/responses, and the sample was considered large and varied enough to describe current practice [19]. An interim analysis was performed after 20 observations. Recruitment of patients stopped after 34 observations.

Directly after the follow-up consultations, the patients were asked to fill in a short questionnaire to assess their satisfaction with information provided by, and communication with, the healthcare provider. The questionnaire was based on the Consumer Quality Index (CQI) for “General practice care” [20] and included questions on patients’ educational attainment, country of origin, and satisfaction with information and communication provided by their healthcare provider. Items on satisfaction (*N* = 5 items) were rated on a four-point Likert scale, ranging from very satisfied (4) to dissatisfied (1). A single multiple-choice question assessed whether patients had received sufficient information about topics such as lifestyle, work, and coping with their disease.

### Data processing

Transcript coding was done in a systematic way by two researchers (LD and JV) who had received training in qualitative research analysis. First, the two researchers independently double coded all transcripts. Individual codes were compared and differences were discussed. The two researchers then grouped the codes into themes. A third researcher (Ms. VvM) was asked to check and comment on all codes to reach a consensus. Detailed records were held of these discussions and decisions, to establish an audit trail. Regular debriefing sessions were held with other members of the research team.

The Verona Coding Definitions of Emotional Sequences (VR-CoDES) were used, a consensus-based system for coding health provider-patient communication sequences in medical consultations [21, 22]. The VR-CoDES consists of two coding manuals, one to code patients’ expressions of emotional distress as cues and concerns (CC) and another to code healthcare provider responses (P). A cue is defined as “a verbal or non-verbal hint which suggests an underlying unpleasant emotion”, whereas a concern is “a clear and unambiguous expression of an unpleasant current or recent emotion” (according to the VR-CoDES-CC manual) [21]. Unpleasant or bothersome symptoms were coded as part of the patients’ cues and concerns. Cues and concerns can be raised spontaneously by the patient (patient elicited), or in response to a topic initiated by the healthcare provider (healthcare provider elicited). Alongside cues and concerns, patients’ questions were also coded. In a few cases, the patient was accompanied by a relative or friend. Questions, cues, and concerns introduced by relatives or friends were coded as part of the patients’ needs.

Responses of healthcare providers to cues and concerns were characterized as either providing or reducing space for further disclosure (according to VR-CoDES-P manual) [22], in which providing space refers to any response that “actively or passively invites or allows the patient to say more about their cue/concern or worry” (including acknowledgement, active invitation, back channeling, showing empathy, and exploration), whereas reducing space refers to any response which “reduces the opportunity for the patient to say more about the cue or concern” (including active blocking, ignoring, information and advice giving, shutting down, and switching topic). More than one response could be given to one cue or concern.

### Data analysis

All the questions, cues, and concerns were categorized into the supportive care domains described by Boyes et al. [23]. The domains represent prevalent supportive care needs of cancer patients in the clinical setting, namely “psychological” (needs related to emotions, coping, and social circles), “health system and information” (obtaining information regarding follow-up schedule, diagnostic test results, side-effects, etc.), “physical and daily living” (physical symptoms, tasks, work and activities, which were further categorized into cancer-related versus unrelated), and “sexuality” (sexual relationships). For this study, the “patient care and support” domain was not included, because it refers to hospital staff acknowledging and showing sensitivity to patients’ feelings and needs, but these were coded as part of the healthcare providers’ responses to cues and concerns.

All the transcripts were coded and analyzed using MAXQDA Plus 2020 Network Software [24]. The questions versus cues and concerns were analyzed and described separately. Member checking was not performed, but the use of the VR-CoDES system will have helped to carefully identify cues, concerns, and responses. Questionnaire data are described using descriptives. The consolidated criteria for reporting qualitative studies (COREQ) were used in this study [25].

## Results

Between September 2013 and November 2014, 34 colon cancer patients were recruited and observed at the outpatient surgery clinic of five Dutch hospitals. Four patients were excluded because the consultation was not part of a routine follow-up visit (*n* = 2), the patient was undergoing active treatment for cancer (*n* = 1), and no audio-tape was available for logistic reasons (*n* = 1). This resulted in a sample size of 30 patients. Of these, 25 patients had a consultation with a surgeon and 5 with a specialized oncology nurse. There were 7 surgeons and 2 specialized nurses who performed these consultations. Overall, the study population included 15 men and 15 women; the mean age was 68.3 (SD 10.1); tumor stages I–III were evenly distributed; and follow-up ranged from 3 to 60 months (Table 1).

**Table 1:** Patient characteristics (categorized by the type of healthcare provider)

*Characteristic*	*Follow-up by a surgeon (n* = *25)*	*Follow-up by a specialized oncology nurse (n* = *5)*
Age in years (mean, SD)	69.5 (10.2)	62.4 (7.9)
Gender, female (*n*)	13	2
Educational attainment (*n*)		
- Primary or none	2	0
- Secondary	10	2
- Vocational education	9	2
- University	1	0
- Unknown	3	1
Tumor stage (*n*)		
- I	7	1
- II–III	16	4
- Unknown	2	NA
Chemotherapy (yes)	6	4
Duration of follow-up in months (median, IQR)	18 (23)	12 (8)

### Patients’ questions

**Fig. 1:**
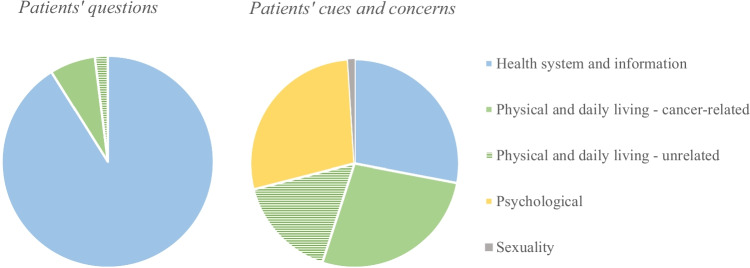
Distribution of patients’ questions, cues, and concerns within the supportive care domains

The 30 patients raised 120 questions, nearly all of which were categorized in the “health system and information” domain (*n* = 110 out of 120; or 92%) (Fig. 1). Most questions related to the planning of follow-up consultations and tests (*n* = 45; 38%). Questions were asked about test results (*n* = 35; 29%), of which most questions related to the level and meaning of carcinoembryonic antigen (CEA) and ultrasound or computed tomography (CT) findings (an example is shown in Table 2, quote 1). Some patients asked about the implications and yield of follow-up testing (*n* = 10; 8%). The patients also asked questions about their medication and side-effects (*n* = 10; 8%), such as how to use their medication to regulate bowel function and how to use preparatory medication for follow-up tests (such as the use of laxatives and narcotics prior to a colonoscopy). Other questions related to patients’ insurance and their wish to receive a copy or statement from the healthcare provider (*n* = 10; 8%).

**Table 2:** Examples of questions, cues and concerns within the supportive care domains

Quote nr	Supportive care domain	
	*Health system and information*	
1	Female, 67 Questions about the value and meaning of the carcinoembryonic antigen (CEA) result	Patient: And the blood test result? Surgeon: The result is 1.8, and last time it was 2.2 Patient: Yes Surgeon: It always varies slightly Patient: Is it better if it’s lower? Surgeon: No, no, that doesn’t make any difference, as long as it is lower than 5.5. The substance is present in blood and can be measured, just like sodium or potassium Patient: Yes Surgeon: Mmm, sometimes it’s a bit higher, sometimes a bit lower
2	Male, 61 Cue miscommunication about the follow-up schedule Response: provide space (acknowledgement)	Patient: I spoke to a colleague of yours on the phone last week about my test results Surgeon: Yes, that’s right, dr. [name]. He made a note of it Patient: And he said to me “you’ll have to come back in six months” and I said “no, that’s not right, I have to come back the 24th for a check-up with dr. [name]” Wife: Then that will be the misunderstanding Surgeon: Yes, I believe so as well
3	Female, 55 Cue relating to the meaning of the carcinoembryonic antigen (CEA) results Response: reduce space (information giving)	Surgeon: The CEA is 3.6 Patient: That’s a bit higher Surgeon: Yes, it’s slightly higher, but that’s not uncommon. It always varies a bit Patient: Varies… yes Surgeon: It should be lower than 5.5, so it’s a good result Patient: Yes Surgeon: It’s not the case, that if it’s slightly higher that there’s more chance that something is wrong Patient: No, exactly, no. It’s the highest level up to now Surgeon: Yes, and next time it might be slightly lower again, so we’ll check it
	*Physical and daily living*	
4	Male, 80 Cue relating to dizziness (complaint unrelated to cancer) Response: provide (exploration) and then reduce space (blocking)	Patient: When I’m in the garden, sitting bending over, I get a bit dizzy when I stand up Surgeon: Is that new or… Patient: No, no Surgeon: You’ve had it before? Patient: Yes Surgeon: It’s nothing to do with your gut, but it is annoying
5	Female, 85 Cue relating to peripheral edema (unrelated problem) Response: provide space (exploration) and then reduce space (information giving)	Patient: Oh yes. Very swollen feet Friend: She’s had them Surgeon: When was that? In August? […] Surgeon: The swelling has nothing to do with your gut but with your heart. Umm, the fluid needs to drain from your legs… umm it’s good that you’ve been given diuretics, to help the process Patient: Yes Surgeon: Yes, but we’ll leave that to the GP
	*Psychological*	
6	Female, 56 Cue relating to a fear of recurrence Response: provide space (acknowledgement)	Patient: Every now and then you have an odd feeling in your body, which everyone has sometimes, then you think: gosh, could this have something to do with it? Surgeon: Yes, I would say that that is a normal reaction of every cancer patient Patient: Yes, yes Surgeon: If you feel something but you have never had anything, then you ignore that feeling. But now you have that reflex: is it cancer?
7	Female, 62 Concern relating to the patients’ wellbeing Response: reduce space (information giving)	Patient: I’m not myself anymore – I’m not the [name] I used to be and that really bothers me Nurse: Yes, but you also need to give it time. You’ve had a tough year and a lot has happened in a short time
	*Sexuality*	
8	Male, 60 Cue relating to ejaculation (unrelated complaint) Response: reduce space (blocking)	Patient: Umm, when I have sex, 99% of the time there are no sperm released. I went to the men’s clinic about this… I was at the men’s clinic and I was given a tablet, but that doesn’t help […] Surgeon: Yes, that fine of course, but it has nothing to do with the cancer

Only a few questions were categorized in the “physical and daily living” domain (*n* = 10; 8%). Three patients asked questions about whether a symptom was related to cancer (i.e., a lump in the skin, stomach ache, and weakened immune system), and two patients inquired about the treatment of an unrelated disease (i.e., hemorrhoids and abdominal aneurysm).

### Patients’ cues and concerns

In total, 420 cues and concerns were identified, of which 315 were patient elicited, and 105 were healthcare provider elicited. Most cues and concerns related to the “physical and daily living” domain (*n* = 181 out of 420; 43%), followed by the “health system and information” (*n* = 119; 28%), and “psychological” domain (*n* = 117; 28%). Few cues and concerns were related to the “sexuality” domain (*n* = 3; 0%) (Fig. 1).

The patients frequently reported symptoms related to colon cancer or its treatment (*n* = 114; 27%), such as changes in bowel habits, fatigue, abdominal pain, flatulence, and fecal blood (this was caused by hemorrhoids). Some patients still found it difficult to resume daily activities and work. The patients who had undergone chemotherapy mentioned symptoms caused by polyneuropathy. The patients often expressed symptoms that were not directly related to cancer (*n* = 67; 16%), such as urological (benign prostatic hyperplasia), musculoskeletal (osteoarthritis), pulmonary (obstructive lung disease), and cardiovascular (congestive heart failure, abdominal aneurysm, and venous insufficiency) symptoms (Table 2, quote 4). Healthcare providers discussed the management of cancer-related problems, whereas the management of unrelated problems was sometimes referred to a relevant specialist (urologist, cardiologist, oncologist) or GP (Table 2, quote 5).

Cues and concerns in the ‘health system and information’ domain often related to miscommunication or a lack of clarity about the follow-up schedule (n = 43; 10%) (Table 2, quote 2). In two cases, the patients were unhappy with the information they had received and did not feel properly informed. Cues and concerns also frequently related to the test results (*n* = 36; 9%). Healthcare providers often provided further information that the test results were normal (Table 2, quote 3). Some patients mentioned problems with their medication and side-effects (*n* = 31; 7%), while others experienced difficulties with their insurance policies (*n* = 9; 2%).

The patients also frequently expressed a fear of recurrence (*n* = 59; 14%) and of needing follow-up testing (because of prior experience or possible complications), and in one instance fear of a late complication of surgery (anastomotic dehiscence) (*n* = 17; 4%). Some patients experienced social difficulties (*n* = 26; 6%), while other reported diminished wellbeing (*n* = 17; 4%) and a loss of personal identity (Table 2, quotes 6 and 7).

Few cues and concerns were expressed in the sexuality domain. One patient mentioned an ejaculation (Table 2, quote 8), but the surgeon actively blocked further discussion of the problem. The specialized nurses, but not the surgeons, used a distress thermometer to evaluate patient burden and possible need of supportive care.

### Healthcare providers’ responses

A total of 464 responses to cues and concerns were identified. Healthcare providers provided space for further disclosure of approximately half of all cues and concerns (*n* = 251 out of 464; or 54%). Table 3 shows the type of response according to the supportive care domains. Space was most often provided to talk about cues and concerns in the “physical and daily living” domain (*n* = 125 out of 202; or 62%), followed by the “psychological” (*n* = 70 out of 133; 53%) and “health system and information” domain (*n* = 56 out of 126; 44%). Providing space for cues and concerns in the physical domain was mostly done explicitly by exploring (*n* = 58 out of 125; 46%) and acknowledging (*n* = 38; 30%) the symptom or problem (supplementary appendix p1 shows all the different response types within each domain). There were no important differences in responses to cancer-related versus unrelated cues and concerns. Reducing space to talk about cues and concerns in the health system and information domain was typically done by providing information and advice (*n* = 59 out of 70; 84%).

**Table 3:** Healthcare providers’ responses to patients’ cues and concerns

Supportive care domain	Responses*	Providing space†	Reducing space†
Health system and information	126	56 (44%)	70 (56%)
Physical and daily living			
- All cues and concerns	202	125 (62%)	77 (38%)
- Cancer-related	123 (61%)	75 (61%)	48 (39%)
- Unrelated	79 (39%)	50 (63%)	29 (37%)
Psychological	133	70 (53%)	63 (47%)
Sexuality	3	NA	3 (100%)
Total	464	251 (54%)	213 (46%)

^*^Since more than one response could be giving to a cue or concern, the total number of responses exceeds the number of cues and concerns

^†^Percentages are calculated based on the number of responses within each domain

### Patients’ satisfaction with care

**Fig. 2:**
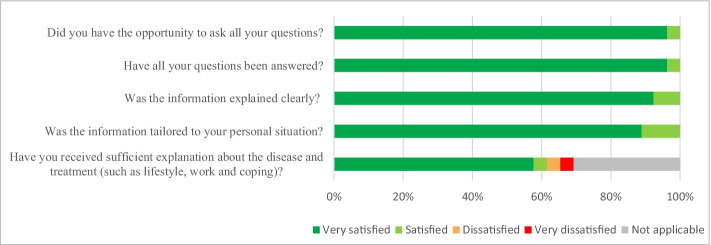
Patient satisfaction with information and communication by their healthcare provider

Of the 30 patients, 27 (90%) returned the questionnaire per email. All the patients were satisfied with the information and communication provided by their healthcare provider (Fig. 2). There were no important differences between patients receiving care from a surgeon versus specialized oncology nurse (data not shown). In the multiple-choice question, three patients indicated that they had received too little information about coping with the disease. Three other patients would have liked to receive more information on cancer and work-related activities.

## Discussion

This multicenter observational study assessed the needs of colon cancer patients expressed during follow-up consultations at an outpatient surgery clinic and how these were addressed by their healthcare providers. Questions typically centered around the health system and information domain, whereas cues and concerns were more often related to the physical and daily living domain, and the psychological domain. Most cues and concerns were related to colon cancer or its treatment, but unrelated cues and concerns were also frequently mentioned. Hardly any questions, cues, and concerns in the sexuality domain were raised by the patients or healthcare providers. Healthcare providers responded to the different types of expressed needs in a similar fashion.

### Comparison to existing literature

The VR-CoDES have been previously used to identify cues and concerns in patients with other types of cancer, who may experience different types of problems during follow-up than those experienced by colon cancer patients [26–28]. The questions, cues, and concerns identified in this study largely overlapped those described in quantitative research [5–7]. Potential problems in the sexuality domain were rarely discussed during consultations, even though it is a prominent need of patients with colon and/or rectal cancer [3, 4]. In addition to the existing literature, this study also investigated healthcare providers’ responses to patients’ cues and concerns. Their responses to cancer-related and (seemingly) unrelated problems were very similar. The nature of the cue or concern therefore does not seem to influence the response. Healthcare providers provided space to talk about approximately half of all cues and concerns (54%). These results are consistent with those of other qualitative observational studies[26, 27], although space was provided less frequently (approximately 40%) in an observational study among cancer survivors in Brazil [28]. A possible explanation for this difference is that we coded unpleasant and bothersome symptoms as part of the patients’ cues and concerns. The difference may also be related to the context and the setting of this particular study. The responses of healthcare providers do not occur at random and can depend on the source, explicitness, and timing of the cue or concern. For example, nurses in admittance interviews are five times more likely to provide space for further disclosure of cues and concerns than oncologists in outpatient follow-up consultations [26]. Limited consultation time, as also seen in this study, may prevent healthcare providers from providing space to talk about cues and concerns.

### GP involvement in cancer survivorship care

Whether or not GPs can play a greater role in colon cancer survivorship care depends on the type of needs expressed by patients during follow-up. In this qualitative observational study, the patients’ questions, cues, and concerns were often related to the planning of follow-up consultations and the meaning of follow-up test results. These types of needs can be answered on the basis of follow-up guidelines and could, in theory, be handled by any healthcare provider, including GPs [18]. Overall, the patients were satisfied with the information and explanations given by their healthcare provider, but some wished for more information about coping with their disease and work-related activities. A recent cross-sectional survey found similar unmet informational needs among colorectal cancer patients [29]. Other healthcare providers may have a different approach to dealing with cues and concerns, and this warrants investigation. GPs are trained to provide holistic care and could be of additional value in addressing some of these aspects during survivorship care. The Institute of Medicine recommends the use of survivorship care plans (SCPs) to improve awareness about cancer treatment and follow-up care, but their impact on health outcomes and healthcare delivery remains unclear [30].

### Recommendations for practice and research

Communication in medical consultations is often dominated by the healthcare provider [31]. We encourage clinicians to discuss all the supportive care domains during follow-up consultations, including potential problems relating to coping and work, as well as sexuality and intimacy. We also encourage clinicians to provide space for further disclosure. Emery et al. have recently published a paper on the management of common clinical problems experienced by cancer survivors [32]. The authors provide examples of ways to initiate conversations about specific problems (e.g., to initiate a conversation about sexuality and intimacy, a clinician could ask; “do you have any concerns about your sex life or sexual function and are these concerns causing you distress?”). Because a single consultation is often not enough to cover all supportive care domains, the authors also suggest the use of screening tools, such as the distress thermometer. These tools can help to prioritize topics and flag potential problems for subsequent consultations. Agenda setting, in which the healthcare provider solicits patients’ agendas, is done infrequently, but can affect the disclosure of concerns/questions [33]. Even asking the patient whether he or she has “some other concerns” instead of “any other concerns”, can reduce the incidence of unmet needs [34]. Application of the VR-CoDES in future studies will facilitate comparative and cross-national research on communication in medical consultations. This can also be used to train physicians in recognizing and managing patients’ emotional distress, by using techniques such as active listening and using open questions [35]. In turn, this may also have a positive effect on patients’ satisfaction with information and communication.

### Strengths and limitations

Studies of cancer patients’ needs are often quantitative and retrospective of nature. Direct observation is suited to study behavior and interaction in its natural setting [36]. This study provides additional evidence by using “real-time” data from routine follow-up consultations at the outpatient clinic, thereby giving a unique perspective on colon cancer patients’ needs and how these are addressed. To our knowledge, this type of qualitative approach to investigate the needs of colon cancer patients has not been previously used.

While we assessed the expressed needs of patients, we did not investigate unexpressed needs. Other methods, both quantitative and qualitative, will be more suitable to address unexpressed needs. Also, the VR-CoDES do not specify non-verbal behavior in detail. Future studies should therefore consider using video-recordings, since audio-recordings do not distinguish non-verbal cues and concerns. Patients’ needs may also change over time, so a longitudinal study design would be of additional value. We stopped data collection when the transcripts provided no new types of questions/cues/concerns/responses, and when the sample was sufficiently large to describe current practice. However, the study included only five consultations with a specialized oncology nurse, so data sufficiency and representativeness may not have been achieved for consultations with these healthcare providers. The difference in consultation time can be largely attributed to differences in the time scheduled for a consultation, with nurses being scheduled 15 min and surgeons 5–10 min. Despite this, the needs expressed by patients did not differ in consultations performed by a surgeon or specialized oncology nurse. That the healthcare providers came from five Dutch hospitals supports the robustness of the findings and reflects the standard of hospital follow-up care. Even though the consultations were performed a few years ago, follow-up practice for colon cancer has changed little since then [18], which is important for the validity of the findings.

## Conclusions

In conclusion, this qualitative observational study improves our understanding of the needs of colon cancer patients expressed during routine follow-up consultations in hospital and how these needs are addressed by healthcare providers. Based on the results, we encourage healthcare providers to discuss all supportive care domains, including potential problems with sexuality and intimacy, and provide space for further disclosure of cues and concerns. GPs are trained in providing holistic care and could play a greater role in addressing patients’ needs.

## Supplementary Information

Below is the link to the electronic supplementary material.

Supplementary file1 (DOCX 33 KB)

## Data Availability

Anonymized data can be made available on request to the corresponding author. The data collected for this study will be stored up to 15 years after the end of study. This time period will take into account possible national and international legal restrictions (i.e., from the Netherlands, E. U.).
