# Supplementary material for: Addressing colon cancer patients’ needs during follow-up consultations at the outpatient clinic: a multicenter qualitative observational study
Source: Support Care Cancer. 2022 Jun 21;30(10):7893–901. doi: 10.1007/s00520-022-07222-z (PMC9512715; doi:10.1007/s00520-022-07222-z)
Supplement: Supplementary file 1 — Supplementary file1 (DOCX 33 KB) [file 520_2022_7222_MOESM1_ESM.docx]

**Supplementary appendix**

Table 1 Healthcare providers’ responses to patients’ supportive care needs.

|  | **Health system and information†** | **Physical and daily living†** | | **Psychological†** | **Sexuality†** |
| --- | --- | --- | --- | --- | --- |
| **Overarching response types** |  | *Cancer-related* | *Unrelated* |  |  |
| Providing space – non-explicit   - Active invitation - Back channel - Implicit empathy | 3 (2%)  NA  5 (4%) | 7 (6%)  10 (8%)  NA | 5 (6%)  4 (5%)  NA | 6 (5%)  8 (6%)  4 (3%) | NA  NA  NA |
| Providing space – explicit   - Exploration - Acknowledgement - Explicit empathy | 21 (17%)  24 (19%)  3 (2%) | 33 (27%)  25 (20%)  NA | 25 (32%)  13 (16%)  3 (4%) | 16 (12%)  26 (20%)  10 (8%) | NA  NA  NA |
| Reducing space – non-explicit   - Ignore - Shutting down | 9 (7%)  NA | 12 (10%)  1 (1%) | 6 (8%)  1 (1%) | 11 (8%)  1 (1%) | NA  NA |
| Reducing space – explicit   - Information and advice - Switching - Active blocking | 59 (47%)  1 (1%)  1 (1%) | 34 (28%)  1 (1%)  NA | 16 (20%)  2 (2%)  4 (5%) | 46 (35%)  5 (4%)  NA | 2 (67%)  NA  1 (33%) |
| **Total responses*** | 126 | 123 | 79 | 133 | 3 |
| * Since more than one response could be giving to a cue or concern, the total number of responses exceeds the number of cues and concerns. † Percentages are calculated based on the number of responses within each domain. | | | | | |
